# Establishment and genetically characterization of patient-derived xenograft models of cervical cancer

**DOI:** 10.1186/s12920-022-01342-5

**Published:** 2022-09-08

**Authors:** Shuangwei Zou, Miaomiao Ye, Jian-an Zhang, Huihui Ji, Yijie Chen, Xueqiong Zhu

**Affiliations:** Department of Obstetrics and Gynecology, The Second Affiliated Hospital of Wenzhou Medical University, No. 109 Xueyuan Xi Road, Wenzhou, 325027 Zhejiang China

**Keywords:** Patient-derived xenograft (PDX), Cervical cancer, Histological analysis, Genetic stability, Somatic mutations, Whole-genome sequencing (WGS), Whole exome sequencing (WES)

## Abstract

**Purpose:**

Patient-derived xenograft (PDX) models were established to reproduce the clinical situation of original cancers and have increasingly been applied to preclinical cancer research. Our study was designed to establish and genetically characterize cervical cancer PDX models.

**Methods:**

A total of 91 fresh fragments obtained from 22 surgically resected cervical cancer tissues were subcutaneously engrafted into female NOD-SCID mice. Hematoxylin and eosin (H&E) staining was performed to assess whether the established PDX models conserved the histological features of original patient cervical cancer tissues. Moreover, a Venn diagram was applied to display the overlap of all mutations detected in whole-genome sequencing (WGS) data from patient original cervical cancer (F0) and F2-, F3-PDX models. The whole exome sequencing (WES) and the “maftools” package were applied to determine the somatic mutations among primary cervical cancers and the established PDX models.

**Results:**

Our study successfully developed a panel of cervical cancer PDX models and the latency time of cervical cancer PDX model establishment was variable with a progressive decrease as the passage number increased, with a mean time to initial growth of 94.71 days in F1 engraftment to 40.65 days in F3 engraftment. Moreover, the cervical cancer PDX models preserved the histological features of their original cervical cancer. WGS revealed that the genome of original cervical cancer was preserved with high fidelity in cervical cancer PDX models throughout the xenografting and passaging process. Furthermore, WES demonstrated that the cervical cancer PDX models maintained the majority somatic mutations of original cervical cancer, of which the KMT2D, LRP1B, NAV3, TP53, FAT1, MKI67 and PKHD1L1 genes were identified as the most frequently mutated genes.

**Conclusions:**

The cervical cancer PDX models preserved the histologic and genetic characteristics of their original cervical cancer, which helped to gain a deeper insight into the genetic alterations and lay a foundation for further investigation of the molecular targeted therapy of cervical cancer.

**Supplementary Information:**

The online version contains supplementary material available at 10.1186/s12920-022-01342-5.

## Introduction

Cervical cancer represents the second leading cause of cancer-related mortality in women globally, with an estimated 604,127 new cases and almost 341,831 deaths occurring in 2020 worldwide [1], which is strongly linked with the persistent infection of high-risk cervical human papillomavirus [2]. The therapeutic options for recurrent/metastatic cervical cancer are still limited, with the main treatment strategy of combining chemotherapy with targeted therapy [3]. Bevacizumab is the only agent approved for targeted therapy of cervical cancer, whose potential clinical benefits have not been sufficiently confirmed, primarily because the patients are not pre-selected for molecular alterations in the targets of interest [4, 5].

Conventional wisdom holds that cancer initiation and progression is attributed to the evolutionary process of genomic rearrangements and somatic mutations [6]. Moreover, genes with high somatic mutation rates are generally considered potential driver genes of cancer [7]. Performing next generation sequencing (NGS) for somatic mutation analysis is prevalent for the identification of critical molecular events involved in tumorigenesis and cancer development [8, 9]. Cervical cancer often harbors activating somatic mutations, including E74 Like ETS Transcription Factor 3 (ELF3) and Core-Binding Factor Subunit Beta (CBFB) genes, and inactivating somatic mutations in the F-Box And WD Repeat Domain Containing 7 (FBXW7), Major Histocompatibility Complex, Class I, B (HLA-B) and E1A Binding Protein P300 (EP300) genes, which may drive oncogenic transformation and chemotherapy resistance of cervical cancer [10]. Therefore, it is of great importance to determine the specific somatic mutations of cervical cancer, which is helpful for the development of precise therapies and targeted therapies for cervical cancer patients.

Patient-derived xenograft (PDX) models are generally constructed by implanting fresh surgical tumor tissues into immune-deficient mice [11], which can be performed orthotopically or heterotopically [12]. Moreover, PDX models maintain the principal molecular characterizations, histological architecture and genomic features of the original cancers [13]. Therefore, in the recent decade, PDX models have been established to reproduce the clinical situation of original cancers [14], and have been increasingly applied in preclinical cancer research [15].

The present study was designed to establish and histologically characterize patient-derived xenograft models of cervical cancer. Moreover, this study implemented Whole-Genome Sequencing (WGS) and Whole Exome Sequencing (WES) to evaluate the genomic features and somatic mutations between cervical cancer PDX models and patients’ original cervical cancer tissues.

## Materials and methods

### Patient specimens

Surgically resected cervical cancer tissues and matched adjacent normal tissues were obtained from cervical cancer patients (*n* = 22) with written informed consents, who underwent radical hysterectomy at the Second Affiliated Hospital of Wenzhou Medical University. The size of each tissue sample measured approximately 1 × 1 × 1 cm. The tissue samples were preserved in ice-cold phosphate-buffered saline (PBS), and transported to the laboratory for processing within 2 h. Furthermore, after removal of necrotic tissues, the resected fresh cervical cancer tissues were sectioned into three sections: one section was prepared for cancer transplantation to establish the PDX models; one section was fixed in 4% paraformaldehyde, and then embedded in paraffin and sectioned for Hematoxylin–eosin (H&E) staining; and the remaining section was flash-frozen with liquid nitrogen for long-term storage at − 80 °C for the further study. The research protocol was ethically reviewed and subsequently approved by the ethics committee of the Second Affiliated Hospital of Wenzhou Medical University.

### Animals

Female NOD-SCID mice, aged four to six weeks, weighed 18–20 g, were purchased from Beijing Vital River Laboratory Animal Technology (Beijing, China). The NOD-SCID mice were housed and maintained in isolator cages under a specific-pathogen-free (SPF) environment with a controlled humidity and temperature, on a standard 12 h light/dark cycle, at Wenzhou Medical University. All animal care and experimental procedures complied with the guidelines for ethical review of animal welfare and were approved by the Institutional Animal Care and Use Committee of Wenzhou Medical University.

### Establishment of cervical cancer PDX models

The cervical cancer tissues from surgery patients (termed as F0) were washed with PBS and subsequently gently cut with sharp, sterile scissors into small pieces of approximately 3 mm^3^. Three to five fragments of minced cervical cancer tissues were aspirated into a 5 mm-trocar sheath individually, which was employed to perform the blunt separation of connective tissue following the cutaneous incision operated in the right buttock of NOD-SCID mice. By pushing the inner core needle of the trocar sheath, the cervical cancer tissues were subcutaneously engrafted into the right buttock of NOD-SCID mice, and the cutaneous incision was sutured with stitches. This generation of NOD-SCID mice harboring patient-derived cervical cancer tissues was termed as F1.

After tumor engraftment, the NOD-SCID mice were monitored for a maximum period of 6 months to confirm whether PDX models were successfully established. If the tumor nodules were not palpable within 6 months, they were considered as engraftment failure. The time interval from engraftment to the presence of palpable tumor was defined as the latency time [16]. By measuring the length and width of the subcutaneous tumor with a calliper weekly, its volume was calculated by the following formula: Volume = 0.5 × (width^2^ × length) [17]. When the volume of the tumor was approximately 1000 mm^3^, the xenograft mice were euthanized by cervical dislocation, and the tumor was excised and sectioned into three sections as the procedure followed for the surgically resected human cervical cancer tissues. Parts of the xenograft tumor were continuously engrafted into new female NOD-SCID mice. Furthermore, the subsequent serially-transplanted xenografts were obtained in a manner similar to that described above, and the tumor engrafted NOD-SCID mice were sequentially termed as F2, F3…Fn respectively.

### Histological analysis

The patient original cervical cancer tissues (F0), F2- and F3-PDX tissues were fixed in 4% paraformaldehyde for 24 h and embedded in paraffin. Tissue slices were sectioned at a thickness of 4 μm and subsequently were stained with hematoxylin and eosin (H&E), which were imaged on an optical microscope. Moreover, the histological comparison among patient original cervical cancer tissues (F0), F2- and F3-PDX tissues was conducted by a senior pathologist based on overall tumor cytoarchitectural features.

### DNA extraction from tissues

The DNeasy Blood and Tissue kit (Qiagen, Hilden, Germany) was applied to extract the DNA from PDX tissues and patient original cervical cancer tissues, which was performed as described in the manufacturer’s instructions.

### Whole-genome sequencing (WGS)

A single patient original cervical cancer (F0, patient id = No.3) and one each of its corresponding cervical cancer PDX models (F2 and F3) were subjected to Whole-Genome Sequencing (WGS) to evaluate their genomic features. The library was constructed by randomly fragmenting the genomic DNA into 350 bp segments. The DNA libraries were high-throughput sequenced using the Illumina HiSeq 4000 platform for paired-end reads of 150 bp. WGS was applied in F0, F2 and F3 at mean coverages of 24.37X, 16.61X and 40.59X, respectively. The analysis of raw-read sequencing data was carried out by taking the human genome build hg19 as the reference genome. Pre-trimming of the DNA sequence was performed by Fastp 0.20.1, and the quality control assessment was carried out with FastQC v0.11.9. The sequence reads were aligned to the human genome GRCh37/hg19 with BWA (version 0.7.17). GATK 3.8–1-0 was conducted for variant calling, and annotation.

### Whole-exome sequencing (WES)

A single patient original cervical cancer (F0, patient id = No.19) and one each of its corresponding cervical cancer PDX models (F2 and F3), and the adjacent noncancerous tissue were subjected to Whole-Exome Sequencing (WES) to identify the somatic mutations among them. For library preparation, the qualified genomic DNA was randomly broken into 180–280 bp fragments. Exonic DNA capture was conducted with Agilent SureSelect Human All ExonV6 (Agilent Technologies) following the manufacturer’s instructions and was subsequently sequenced using the Illumina NovaSeq 6000 platform for paired-end reads of 150 bp. WES was performed on F0, F2, F3 and adjacent noncancerous tissues at mean coverages of 74.41X, 63.90X, 69.87X and 90.16X, respectively. The analysis of raw-read sequencing data was carried out by taking the human genome build hg19 as reference genome. Pre-trimming of DNA sequence was performed by Fastp 0.20.1, and the quality control assessment was carried out with FastQC v0.11.9. The sequence reads were aligned to the human genome GRCh37/hg19 with BWA (version 0.7.17). GATK 3.8-1-0 was conducted for variant calling, and annotation.

### Somatic mutation analysis

The somatic mutation data were annotated and converted to the Mutation Annotation Format (MAF), and the “maftools” R package was applied to screen the differentially mutated genes in F0, F2 and F3 - PDX tissues [18]. And we have removed outliers by setting rmOutlier = T option when using plotmafSummary function. Moreover, TCGA somatic exomic mutations of 289 cervical cancer samples were acquired from The Cancer Genome Atlas (TCGA) portal (https://portal.gdc.cancer.gov/) [19].

### Data availability

The raw data of WGS and WES were submitted to the Baidu Netdisk (https://pan.baidu.com/s/1X7KKnt8he2GXiM1X0FADhg), with the dataset code: h69i.

### Statistical analysis

SPSS Statistics Version 25 (IBM Corp., Armonk, NY, USA) was applied for the statistical analyses. The normality of variables was assessed by the Shapiro–Wilk normality test. Normally and non-normally distributed variables were described as mean ± SD and median (*P*25, *P*75), respectively. Qualitative variables were described as numbers and percentages. Non-normally distributed data were analyzed with the nonparametric Mann–Whitney U-test. The rates were compared using the Chi-square test. *P* < 0.05 was considered statistically significant.

## Results

### Establishment and validation of cervical cancer PDX models

Surgically resected primary cervical cancer tissues were collected from twenty-two patients to establish cervical cancer PDX models, and the tissues were histopathologically confirmed as cervical squamous cancer. In more detail, fragments of twenty-two primary cervical cancer tissues were engrafted into ninety-one female NOD-SCID mice aged six to eight weeks (Additional file 1: Table S1), among which twenty-eight F1 mice formed tumors. Therefore, the F1-PDX engraftment success rate was 30.77%, with a median latency time being 87.00 (77.25,104.00) days. Six of twenty-eight F1-PDX died in the process of tumor growth, with a mortality of 21.43%. Moreover, the specific information of F2- and F3-PDX were shown in the Table 1. The differences among latency time to establish F1-PDX, F2-PDX and F3-PDX were statistically significant. Furthermore, statistically significant differences were found among success rates of developing F1-PDX, F2-PDX and F3-PDX. The mortality between F1- and F2-PDX, F1-and F3-PDX, F2- and F3-PDX showed no statistical difference. Unfortunately, cryopreserved F0 cervical cancer tissues were implanted into NOD-SCID mice, showing a 0% engraftment success rate.

**Table 1:** The establishment of subcutaneous cervical cancer—PDX^*^ models

PDX models	Failure (%)	Success (%)	Latency time (days)^#^	Mortality^a^ (%)
F1 engraftment results	63 (69.23)	28 (30.77)	87.00 (77.25,104.00)	6 (21.43)
F2 engraftment results	17 (31.48)	37 (68.52)	48.00 (40.00,60.50)	5 (13.51)
F3 engraftment results	7 (12.07)	51 (87.93)	38.00 (29.00,46.00)	5 (9.80)

Data are presented as n (%) or median (P25, P75)

*Patient - derived xenograft (PDX)

^#^The time interval from engraftment to the presence of palpable tumor was defined as the latency time

^a^The mortality of mice among the successful cervical cancer engrafted mice

**Fig. 1:**
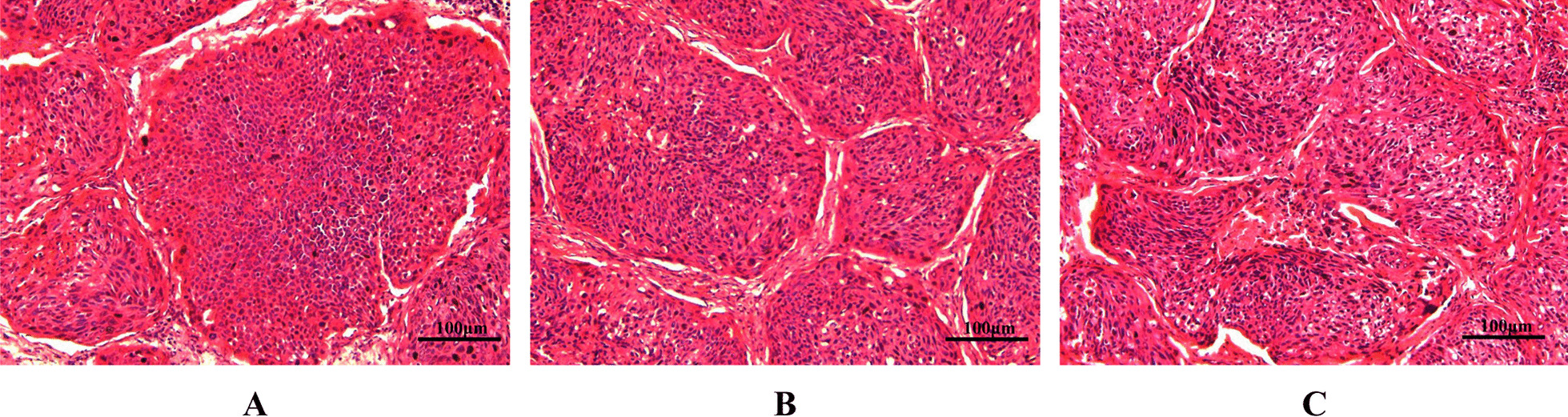
The hematoxylin and eosin (H&E) staining of the patient original cervical cancer and the corresponding cervical cancer-PDX models. **A** Representative images of cervical cancer (F0). **B** Representative images of F2-PDX models. **C** Representative images of F3-PDX models. Micrographs were captured under 200-fold magnification

Histological analysis was performed to assess whether the established PDX conserved the histological features of patient original cervical cancer tissues. H&E staining of cancer tissues from F2- and F3-PDX illustrated that the cytoarchitectural characteristics were similar to those of the patient original cervical cancer tissues morphologically, with the representative micrographs showing in Fig. 1.

### Genomic features retained during PDX tumor passages

**Fig. 2:**
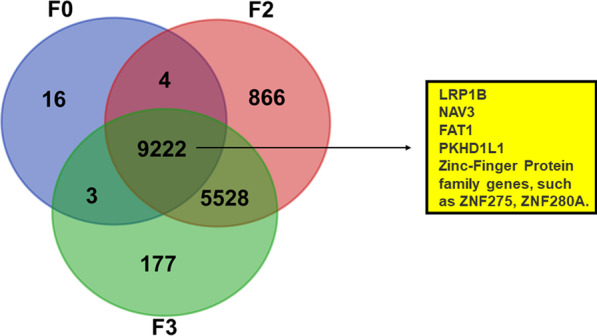
Venn diagram exhibiting the overlap of all mutations detected in whole-genome sequencing data from patient original cervical cancer (F0) and F2-, F3-PDX models

The whole-genome sequencing (WGS) was performed to evaluate the genome-wide mutations between a single patient original cervical cancer (F0, patient id = No.3) and one each of its corresponding cervical cancer PDX models (F2 and F3). A total of 9222 overlapping mutations were found among patient original cervical cancer (F0) and F2-, F3-PDX, of which including the Zinc-Finger Protein family genes, such as ZNF275 and ZNF280A. Moreover, 9226 and 9225 mutations in original cervical cancer were also found in the corresponding F2-PDX and F3-PDX respectively, which accounted for 99.79% and 99.78% of the mutations in the original cervical cancer. Therefore, the cervical cancer PDX models maintained the genetic characteristics and retained most of the mutations of original cervical cancer throughout the xenografting and passaging process, and the detailed results were presented in Fig. 2.

### Somatic mutational status of cervical cancer and PDX models

**Fig. 3:**
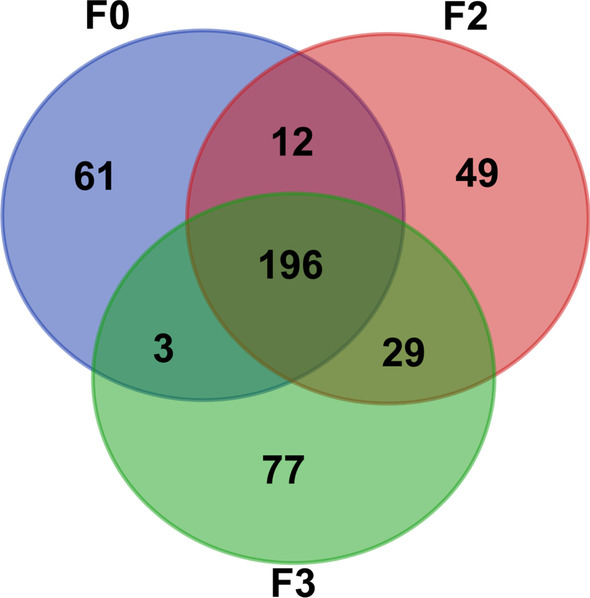
Venn diagram exhibiting the overlap of all somatic mutations detected in whole-exome sequencing data from patient original cervical cancer (F0) and F2-, F3-PDX models

The whole-exome sequencing (WES) was performed to identify somatic mutations in one single patient original cervical cancer (F0, patient id = No.19) and one each of its corresponding cervical cancer PDX models (F2 and F3). When compared with the somatic mutations in the adjacent noncancerous tissues, the original cervical cancer (F0), F2- and F3-PDX samples exhibited 196 overlapping somatic mutations, with 272 somatic mutations in F0, 286 somatic mutations in F2-PDX, and 315 somatic mutations in F3-PDX (Fig. 3).

**Fig. 4:**
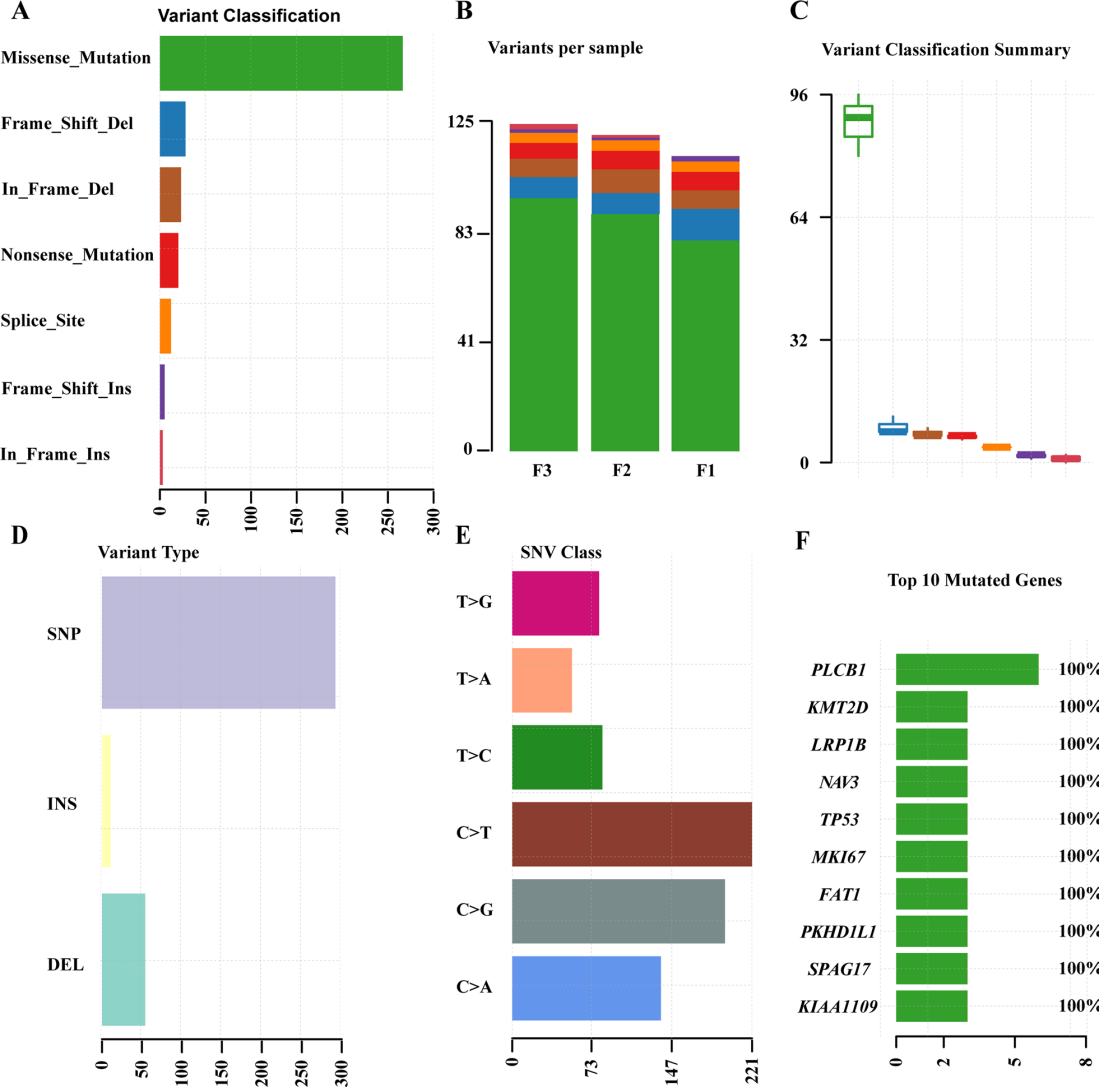
Landscape of the somatic mutation profiles in original cervical cancer (F0), F2-PDX and F3-PDX models based on the whole-exome sequencing. **A** Bundled bar chart illustrating the top seven variant classifications. Green: missense mutation; Blue: frame shift del; Brown: in frame del; Red: nonsense mutation; Orange: splice site; Purple: frame shift ins; Dark red: in frame ins. The same annotation was applied in (**B**) and (**C**). **B** Histogram revealing the variants number in each sample. **C** Box plots showing the summary of variant classifications. The Y-axis represented the mean values with standard deviations of the somatic variant counts in original cervical cancer (F0), F2- and F3-PDX samples. **D** Bundled bar chart presenting the variant types. **E** Bundled bar chart displaying the single nucleotide variant (SNV) classifications. **F** Horizontal histogram exhibiting the top 10 mutated genes in all samples. The X-axis of (**A**), (**D**), (**E**), (**F**) and the Y-axis of (**B**) represented the somatic variant counts. SNP, single-nucleotide polymorphism. INS, insert. DEL, deletion. SNV, single-nucleotide variant

Altogether, these somatic mutations were further characterized and represented in different categories by conducting “maftools” analysis (Fig. 4). In particular, the “maftools” analysis displayed the top seven variant classifications in original cervical cancer (F0), F2- and F3-PDX samples, of which the missense mutation was the most frequently observed variant classification (Fig. 4A). Moreover, the somatic variant counts of missense mutation, frame shift del, in frame del, nonsense mutation, splice site, frame shift ins and in frame ins in separately original cervical cancer (F0), F2- and F3-PDX samples were shown in Fig. 4B. Figure 4C exhibited the mean values with standard deviations of the counts of these seven variants in the original cervical cancer (F0), F2- and F3-PDX samples. The single nucleotide polymorphism (SNP) was the most abundant mutation type (Fig. 4D). Meanwhile, the C > T variant was the most common type that observed in the single nucleotide variant (SNV) classification (Fig. 4E). In addition, the horizontal histogram exhibited the top ten mutated genes with 100% mutation rates, which implied that these ten genes were mutated in all the F0, F2- and F3-PDX samples (Fig. 4F and Additional file 2: Table S2). These top ten mutated genes included phospholipase C beta 1 (PLCB1), lysine methyltransferase 2D (KMT2D), low-density lipoprotein receptor-related protein 1B (LRP1B), neuron navigator 3(NAV3), tumor protein P53 (TP53), marker of proliferation Ki-67 (MKI67), FAT atypical cadherin 1 (FAT1), PKHD1 like 1 (PKHD1L1), sperm associated antigen 17 (SPAG17) and KIAA1109, which had different counts of somatic mutations (Fig. 4F).

**Fig. 5:**
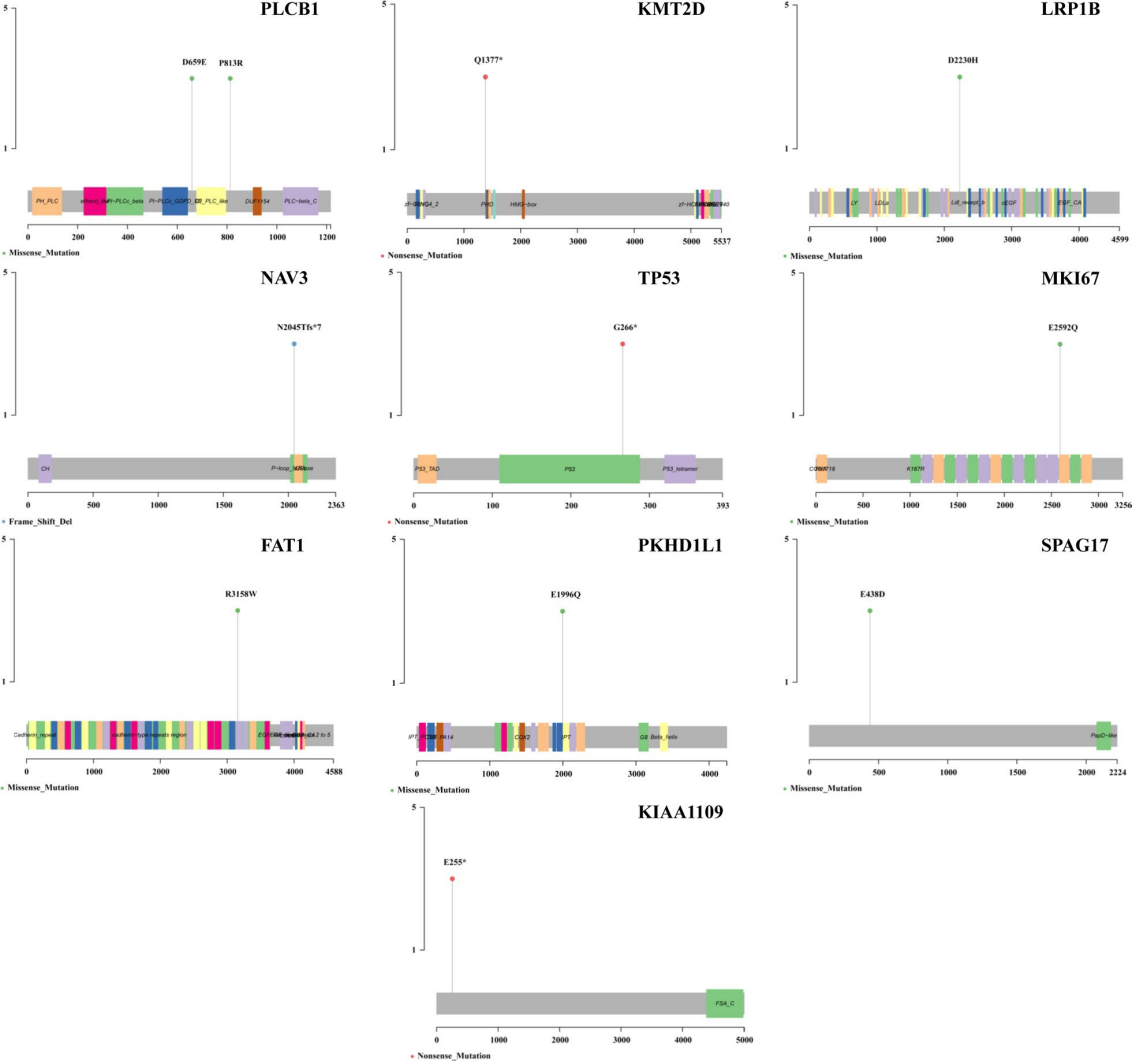
Lollipop plot exhibiting the distribution of mutations of the ten frequently mutated genes

The distribution of mutations of these ten frequently mutated genes was exhibited by a Lollipop plot (Fig. 5). D659E and P813R were the most common missense mutations in PLCB1. Q1377*, G266* and E255* were the most frequent nonsense mutations in KMT2D, TP53 and KIAA1109, respectively. D2230H, R3158W, E2592Q, E1996Q and E438D were the most common missense mutations in LRP1B, FAT1, MKI67, PKHD1L1 and SPAG17, respectively. N2045Tfs*7 was the most frequent frameshift deletions in NAV3. In addition, the Variant Allele Frequency (VAF) information of these ten representative genes identified by WES in patient original cervical cancer (F0) and cervical cancer PDX models (F2 and F3) was displayed in Table 2.

**Table 2:** The variant allele frequency (VAF) information of the representative mutations in patient original cervical cancer (F0) and cervical cancer PDX models (F2 and F3)

Gene-symbol	Variant type			cDNA			Amino acid			VAF		
	F0	F2	F3	F0	F2	F3	F0	F2	F3	F0	F2	F3
PLCB1	SNP			c.1977C > G			p. D659E			41.54%	58.93%	47.30%
	SNP			c.2438C > G			p. P813R			35.09%	42.86%	41.51%
KMT2D	SNP			c.4129C > T			p. Q1377*			25.00%	26.92%	37.50%
LRP1B	SNP			c.6688G > C			p. D2230H			26.67%	33.33%	40.00%
NAV3	DEL			c.6134del			p. N2045Tfs*7			23.53%	52.63%	44.44%
TP53	SNP			c.796G > T			p. G266*			69.57%	100.00%	100.00%
MKI67	SNP			c.7774G > C			p. E2592Q			61.90%	96.55%	100.00%
FAT1	SNP			c.9472C > T			p. R3158W			51.61%	97.22%	100.00%
PKHD1L1	SNP			c.5986G > C			p. E1996Q			18.42%	29.58%	29.73%
SPAG17	SNP			c.1314G > C			p. E438D			31.82%	42.86%	57.14%
KIAA1109	SNP			c.763G > T			p. E255*			72.41%	92.31%	100.00%

**Fig. 6:**
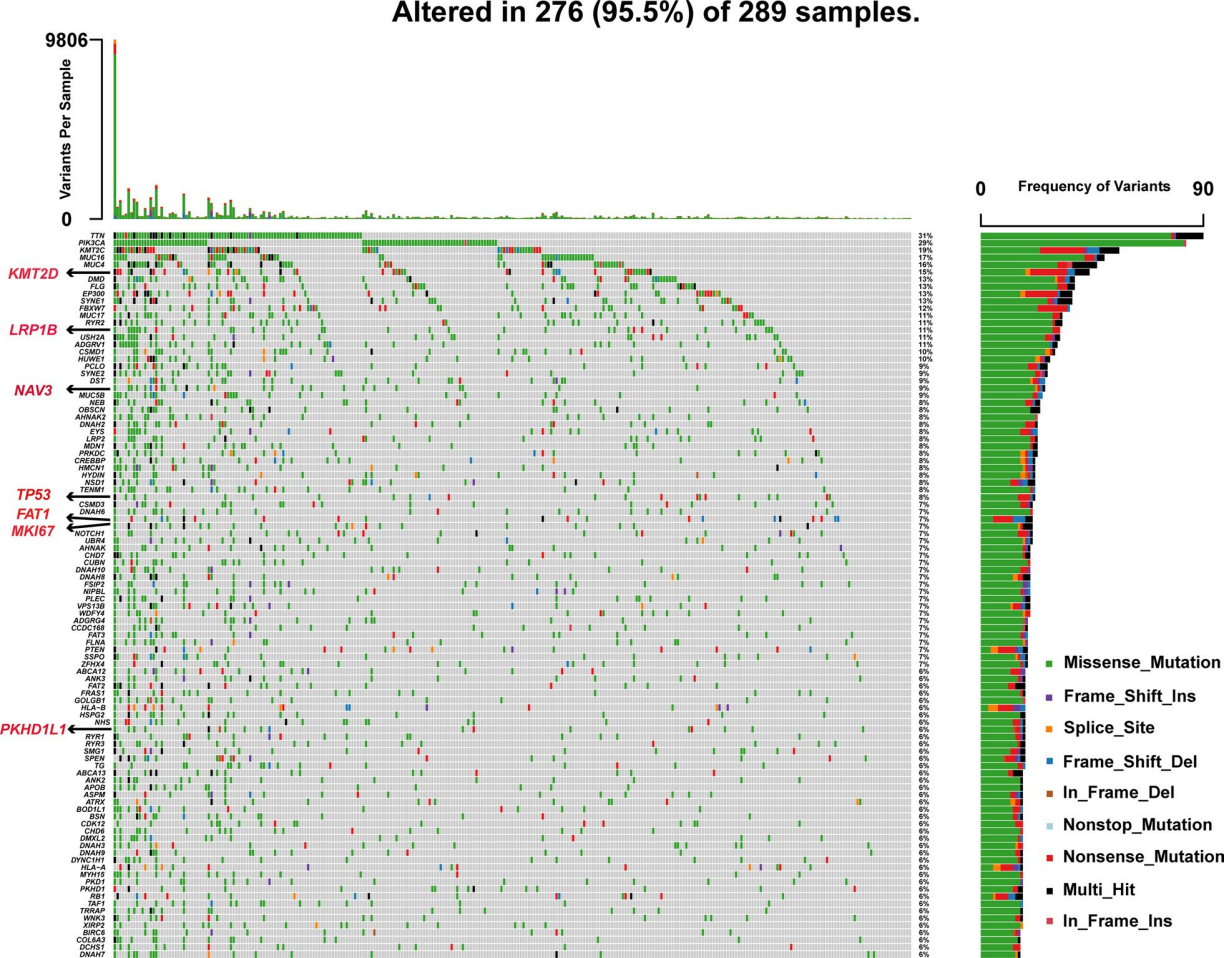
Landscape of somatic mutation profiles in 289 cervical cancer samples based on the TCGA database. The number of variants in each sample was displayed in the top panel. The mutation types were described with diverse colors in the bottom panel. The mutation frequency of each gene in all samples was revealed in barplot in the right panel

Furthermore, the somatic mutated genes recognized using WES in original cervical cancer, F2-PDX and F3-PDX were next confirmed in the TCGA database. The seven somatic mutated genes (KMT2D, LRP1B, NAV3, TP53, MKI67, FAT1, PKHD1L1) identified by WES were part of the top 100 mutated genes in cervical cancer samples, which were highlighted in the waterfall plot obtained from the TCGA database (Fig. 6).

## Discussion

Constructing an ideal animal model for studying cervical cancer is essential for translational cancer research. Previous studies have displayed different protocols to establish cervical cancer PDX models, of which the engraftment success rates varied from 0 to 75% and the latency time fluctuated between 10 days and 12 months [20–24]. In our current study, we successfully developed a panel of cervical cancer PDX models by subcutaneously inoculating fresh cervical cancer tissues into NOD-SCID mice, which preserved the histologic characteristics of original cervical cancer. The latency time of cervical cancer PDX model establishment was variable with a progressive decrease as the passage number increased, with a mean time to initial growth of 94.71 days in F1 engraftment to 40.65 days in F3 engraftment. Additionally, the increased engraftment success rates of cervical cancer PDX models were revealed in our study during serial transplantation, with the success rate of F3 engraftment reaching 87.93%.

Furthermore, the next generation sequencing (NGS) provided a comprehensive approach to identify genomic biomarkers with high fidelity between the cervical cancer PDX models and the patients’ original cervical cancer tissues, which potentially offers a molecular basis for clinical targeted therapy. Our study revealed that most genomic features of original cervical cancer were retained in cervical cancer PDX models throughout the xenografting and passaging process by performing WGS. And the cervical cancer PDX models maintained the majority of somatic mutations of their original cervical cancer, which was confirmed by WES. Full concordance in representative mutated genes, including KMT2D, LRP1B, NAV3, TP53, FAT1, MKI67 and PKHD1L1, was observed in original cervical cancer, F2-PDX models and F3-PDX models, which were also identified as frequently mutated genes in cervical cancer samples in the TCGA database. Largely compatible with previous researches [25–28], our study also identified genetic mutations of TP53 and MKI67 in the cervical cancer. In addition, our study firstly revealed the somatic mutations of KMT2D, NAV3 and PKHD1L1 in cervical cancer, to our knowledge. There are limited researches that reported the mutations of LRP1B and FAT1 genes in cervical cancer previously.

Low-density lipoprotein receptor-related protein 1B (LRP1B), which is localized on chromosome 2q, encodes an endocytic LDL-family receptor [29]. The LRP1B gene is considered as a putative tumor suppressor, and mutations in LRP1B are frequently observed in many cancers [30, 31]. Based on the whole-genome sequencing and high-throughput viral integration detection of HPV integration in cervical cancer, Hu et al. [32] pointed out that LRP1B was identified as an HPV integration hotspot in cervical cancer, and the downregulated expression of LRP1B protein was found when HPV integrated in LRP1B introns, which potentially become a novel biomarker for early screening, diagnosis and personalized therapy of HPV integration-driven cervical cancer. Consistently, Cao et al. [33] stated that LRP1B mutation was correlated with HPV 16 integration status, and HPV-LRP1B integration decreased the expression of LRP1B, which was obtained from mining TCGA database. Moreover, this study also indicated that LRP1B mutation was in a relationship with unfavorable outcomes of cervical cancer patients [33]. In our present study, the missense mutation of LRP1B was detected in original cervical cancer, F2-PDX models and F3-PDX models, which may potentially become a new therapeutic target for cervical cancer patients, and further study will be needed to discover a novel and efficient targeted therapy.

FAT atypical cadherin 1 (FAT1) is a member of the family of fat-like atypical cadherins that encodes 4588 amino acid residues [34, 35]. The mutations in FAT1 gene were detected frequently in diverse cancers, particularly in squamous cell carcinomas, potentially serving as an oncogene or a tumor suppressor [36, 37]. Reportedly, inactivating mutations in the FAT1 gene potentially activated the Wnt signaling pathway and promoted the carcinogenesis, such as in head and neck squamous cell carcinomas [38]. Moreover, Chung et al. [39] performed the WES of 15 cervical adenocarcinoma-normal pairs and indicated that FAT1 was the most significantly mutated gene in cervical adenocarcinoma, with missense and nonsense mutations. Huang et al. [40] conducted the WGS or WES of 102 cervical cancer-normal pairs, and the mutational analysis discovered that the FAT1 was frequently mutated in cervical cancer, which accounting for 8.8% of all cases (9/102). In our current research, the missense mutation of FAT1 was identified in original cervical cancer, F2-PDX models and F3-PDX models. Therefore, the cervical cancer PDX models conserved the somatic mutations of FAT1 in original cervical cancer, and the mutated FAT1 gene could be utilized as the molecular target of new therapeutic regimens for cervical cancer patients.

## Conclusion

In summary, we succeeded in generating serially transplantable cervical cancer PDX models by subcutaneously inoculating fresh cervical cancer tissues into NOD-SCID mice, which exhibited a high concordance of histopathology and genome of the primary cervical cancer patient tissues. Performing WES in original cervical cancer tissues and cervical cancer PDX models helped to gain a deeper insight into the genetic alterations that were essential for the carcinogenesis of cervical cancer. Our established cervical cancer PDX models combined with genomic annotations can be employed to explore the novel molecular targeted therapies, validate novel therapeutic regimens, and predict the sensitivity of chemotherapeutic drug in cervical cancer patients in the future. Therefore, the present study lays a foundation for further investigation of the personalized treatments of cervical cancer patients and has important implications for translational cancer research.

## Supplementary Information

**Additional file 1**. **Table S1**. The data of our established cervical cancer PDX lineages.

**Additional file 2**. **Table S2**. The somatic mutation information of the top ten mutated genes.

## Data Availability

The raw data of WGS and WES were submitted to the Baidu Netdisk (https://pan.baidu.com/s/1X7KKnt8he2GXiM1X0FADhg), with the dataset code: h69i.

## Abbreviations

PDX: Patient-derived xenograft
H&E: Hematoxylin and Eosin
NGS: Next generation sequencing
WGS: Whole-genome sequencing
WES: Whole exome sequencing
ELF3: E74 Like ETS transcription factor 3
CBFB: Core-binding factor subunit beta
FBXW7: F-Box and WD repeat domain containing 7
HLA-B: Major histocompatibility complex, class I, B
EP300: E1A binding protein P300
PBS: Phosphate-buffered saline
SPF: Specific-pathogen-free
MAF: Mutation annotation format
TCGA: The cancer genome atlas
SNP: Single nucleotide polymorphism
PLCB1: Phospholipase C beta 1
KMT2D: Lysine methyltransferase 2D
LRP1B: Low-density lipoprotein receptor-related protein 1B
NAV3: Neuron navigator 3
TP53: Tumor protein P53
MKI67: Marker of proliferation Ki-67
FAT1: FAT atypical cadherin 1
PKHD1L1: PKHD1 Like 1
SPAG17: Sperm associated antigen 17

## Declarations

### Statement of ARRIVE guidelines

Our study is reported in accordance with ARRIVE guidelines.

## References

[1] Sung H, Ferlay J, Siegel R, Laversanne M, Soerjomataram I, Jemal A (2021). Global cancer statistics 2020: GLOBOCAN estimates of incidence and mortality worldwide for 36 cancers in 185 countries. CA Cancer J Clin.

[2] Allouch S, Malki A, Allouch A, Gupta I, Vranic S, Al Moustafa AE (2020). High-risk HPV oncoproteins and PD-1/PD-L1 interplay in human cervical cancer: recent evidence and future directions. Front Oncol.

[3] Choi CH, Choi HJ, Lee JW, Kang ES, Cho D, Park BK (2020). Phase I study of a B cell-based and monocyte-based immunotherapeutic vaccine, BVAC-C in human papillomavirus type 16- or 18-positive recurrent cervical cancer. J Clin Med.

[4] Martinho O, Silva-Oliveira R, Cury FP, Barbosa AM, Granja S, Evangelista AF (2017). HER family receptors are important theranostic biomarkers for cervical cancer: blocking glucose metabolism enhances the therapeutic effect of HER inhibitors. Theranostics.

[5] Roszik J, Ring KL, Wani KM, Lazar AJ, Yemelyanova AV, Soliman PT (2018). Gene expression analysis identifies novel targets for cervical cancer therapy. Front Immunol.

[6] Klco JM, Spencer DH, Miller CA, Griffith M, Lamprecht TL, O'Laughlin M (2014). Functional heterogeneity of genetically defined subclones in acute myeloid leukemia. Cancer Cell.

[7] Zhao S, Liu J, Nanga P, Liu Y, Cicek AE, Knoblauch N (2019). Detailed modeling of positive selection improves detection of cancer driver genes. Nat Commun.

[8] Lu J, Ding Y, Chen Y, Jiang J, Chen Y, Huang Y (2021). Whole-exome sequencing of alpha-fetoprotein producing gastric carcinoma reveals genomic profile and therapeutic targets. Nat Commun.

[9] Schrader KA, Cheng DT, Joseph V, Prasad M, Walsh M, Zehir A (2016). Germline variants in targeted tumor sequencing using matched normal DNA. JAMA Oncol.

[10] Crowley FJ, O'Cearbhaill RE, Collins DC (2021). Exploiting somatic alterations as therapeutic targets in advanced and metastatic cervical cancer. Cancer Treat Rev.

[11] Takada K, Aizawa Y, Sano D, Okuda R, Sekine K, Ueno Y (2021). Establishment of PDX-derived salivary adenoid cystic carcinoma cell lines using organoid culture method. Int J Cancer.

[12] Landuzzi L, Manara MC, Lollini PL, Scotlandi K (2021). Patient derived xenografts for genome-driven therapy of osteosarcoma. Cells.

[13] Koga Y, Ochiai A (2019). Systematic review of patient-derived xenograft models for preclinical studies of anti-cancer drugs in solid tumors. Cells.

[14] He S, Hu B, Li C, Lin P, Tang WG, Sun YF (2018). PDXliver: a database of liver cancer patient derived xenograft mouse models. BMC Cancer.

[15] Wu CX, Wang XQ, Chok SH, Man K, Tsang SHY, Chan ACY (2018). Blocking CDK1/PDK1/beta-Catenin signaling by CDK1 inhibitor RO3306 increased the efficacy of sorafenib treatment by targeting cancer stem cells in a preclinical model of hepatocellular carcinoma. Theranostics.

[16] Zhao L, Chen H, Guo Z, Fu K, Yao L, Fu L (2020). Targeted radionuclide therapy in patient-derived xenografts using (177)Lu-EB-RGD. Mol Cancer Ther.

[17] Tew BY, Legendre C, Schroeder MA, Triche T, Gooden GC, Huang Y (2020). Patient-derived xenografts of central nervous system metastasis reveal expansion of aggressive minor clones. Neuro Oncol.

[18] Mayakonda A, Lin DC, Assenov Y, Plass C, Koeffler HP (2018). Maftools: efficient and comprehensive analysis of somatic variants in cancer. Genome Res.

[19] Hutter C, Zenklusen JC (2018). The cancer genome atlas: creating lasting value beyond its data. Cell.

[20] Hoffmann C, Bachran C, Stanke J, Elezkurtaj S, Kaufmann AM, Fuchs H (2010). Creation and characterization of a xenograft model for human cervical cancer. Gynecol Oncol.

[21] Chaudary N, Pintilie M, Schwock J, Dhani N, Clarke B, Milosevic M (2012). Characterization of the tumor-microenvironment in patient-derived cervix xenografts (OCICx). Cancers.

[22] Hiroshima Y, Zhang Y, Zhang N, Maawy A, Mii S, Yamamoto M (2015). Establishment of a patient-derived orthotopic Xenograft (PDOX) model of HER-2-positive cervical cancer expressing the clinical metastatic pattern. PLoS ONE.

[23] Oh D-Y, Kim S, Choi Y-L, Cho YJ, Oh E, Choi J-J (2015). HER2 as a novel therapeutic target for cervical cancer. Oncotarget.

[24] Larmour LI, Cousins FL, Teague JA, Deane JA, Jobling TW, Gargett CE (2018). A patient derived xenograft model of cervical cancer and cervical dysplasia. PLoS ONE.

[25] Tornesello ML, Buonaguro L, Buonaguro FM (2013). Mutations of the TP53 gene in adenocarcinoma and squamous cell carcinoma of the cervix: a systematic review. Gynecol Oncol.

[26] Tommasino M, Accardi R, Caldeira S, Dong W, Malanchi I, Smet A (2003). The role of TP53 in cervical carcinogenesis. Hum Mutat.

[27] Yu L, Fei L, Liu X, Pi X, Wang L, Chen S (2019). Application of p16/Ki-67 dual-staining cytology in cervical cancers. J Cancer.

[28] Krtinic D, Zivadinovic R, Jovic Z, Pesic S, Mihailovic D, Ristic L (2018). Significance of the Ki-67 proliferation index in the assessment of the therapeutic response to cisplatin-based chemotherapy in patients with advanced cervical cancer. Eur Rev Med Pharmacol Sci.

[29] Wu CC, Rau KM, Lee WC, Hsieh MC, Liu JS, Chen YY (2019). Presence of chronic obstructive pulmonary disease (COPD) impair survival in lung cancer patients receiving epidermal growth factor receptor-tyrosine kinase inhibitor (EGFR-TKI): a nationwide, population-based cohort study. J Clin Med.

[30] Wang M, Xiong Z (2021). The mutation and expression level of LRP1B are associated with immune infiltration and prognosis in hepatocellular carcinoma. Int J Gen Med.

[31] Hu S, Zhao X, Qian F, Jin C, Hou K (2021). Correlation between LRP1B mutations and tumor mutation burden in gastric cancer. Comput Math Methods Med.

[32] Hu Z, Zhu D, Wang W, Li W, Jia W, Zeng X (2015). Genome-wide profiling of HPV integration in cervical cancer identifies clustered genomic hot spots and a potential microhomology-mediated integration mechanism. Nat Genet.

[33] Cao CH, Liu R, Lin XR, Luo JQ, Cao LJ, Zhang QJ (2021). LRP1B mutation is associated with tumor HPV status and promotes poor disease outcomes with a higher mutation count in HPV-related cervical carcinoma and head & neck squamous cell carcinoma. Int J Biol Sci.

[34] Helmbacher F (2018). Tissue-specific activities of the Fat1 cadherin cooperate to control neuromuscular morphogenesis. PLoS Biol.

[35] Lin SC, Lin LH, Yu SY, Kao SY, Chang KW, Cheng HW (2018). FAT1 somatic mutations in head and neck carcinoma are associated with tumor progression and survival. Carcinogenesis.

[36] Li M, Zhong Y, Wang M (2021). Fat1 suppresses the tumor-initiating ability of nonsmall cell lung cancer cells by promoting Yes-associated protein 1 nuclear-cytoplasmic translocation. Environ Toxicol.

[37] Mengyue Chen XS, Yanzhou W, Kaijian L, Cheng C, Xiongwei C, Xiaolong L, Zhiqing L (2019). FAT1 inhibits the proliferation and metastasis of cervical cancer cells by binding β-catenin. Int J Clin Exp Pathol.

[38] Morris LG, Kaufman AM, Gong Y, Ramaswami D, Walsh LA, Turcan S (2013). Recurrent somatic mutation of FAT1 in multiple human cancers leads to aberrant Wnt activation. Nat Genet.

[39] Chung TK, Van Hummelen P, Chan PK, Cheung TH, Yim SF, Yu MY (2015). Genomic aberrations in cervical adenocarcinomas in Hong Kong Chinese women. Int J Cancer.

[40] Huang J, Qian Z, Gong Y, Wang Y, Guan Y, Han Y (2019). Comprehensive genomic variation profiling of cervical intraepithelial neoplasia and cervical cancer identifies potential targets for cervical cancer early warning. J Med Genet.

