# Supplementary material for: Establishment and genetically characterization of patient-derived xenograft models of cervical cancer
Source: BMC Med Genomics. 2022 Sep 8;15:191. doi: 10.1186/s12920-022-01342-5 (PMC9461207; doi:10.1186/s12920-022-01342-5)
Supplement: Supplementary file 1 — Additional file 1. Table S1. The data of our established cervical cancer PDX lineages. [file 12920_2022_1342_MOESM1_ESM.docx]

| Supplementary table 1: The data of our established cervical cancer PDX lineages. | | | |
| --- | --- | --- | --- |
| Patient-id (F0) | F1 engraftment results | F2 engraftment results | F3 engraftment results |
| No.1 | No.1-F1-1-success | No.1-F2-1-success | No.1-F3-1-success |
|  |  | No.1-F2-2-failure | / |
|  | No.1-F1-2-success | No.1-F2-1-success | No.1-F3-1-success |
|  | No.1-F1-3-failure | / | / |
|  | No.1-F1-4-failure | / | / |
| No.2 | No.2-F1-1-success | No.2-F2-1-success | No.2-F3-1-success |
|  |  | No.2-F2-2-failure | / |
|  |  | No.2-F2-3-failure | / |
|  | No.2-F1-2-failure | / | / |
|  | No.2-F1-3-failure | / | / |
|  | No.2-F1-4-failure | / | / |
| No.3^*^ | No.3-F1-1-success | No.3-F2-1-success^*^ | No.3-F3-1-success^*^ |
|  |  |  | No.3-F3-2-success |
|  | No.3-F1-2-success | No.3-F2-1-success | No.3-F3-1-success |
|  |  | No.3-F2-2-success | No.3-F3-1-success |
|  | No.3-F1-3-failure | / | / |
|  | No.3-F1-4-failure | / | / |
|  | No.3-F1-5-failure | / | / |
| No.4 | No.4-F1-1-success | No.4-F2-1-success | No.4-F3-1-success |
|  |  |  | No.4-F3-2-success |
|  |  | No.4-F2-2-success | No.4-F3-1-success |
|  |  | No.4-F2-3-failure | / |
|  | No.4-F1-2-failure | / | / |
|  | No.4-F1-3-failure | / | / |
|  | No.4-F1-4-failure | / | / |
|  | No.4-F1-5-failure | / | / |
| No.5 | No.5-F1-1-success | No.5-F2-1-success | No.5-F3-1-success |
|  |  |  | No.5-F3-2-success |
|  |  |  | No.5-F3-3-failure |
|  |  | No.5-F2-2-failure | / |
|  | No.5-F1-2-failure | / | / |
|  | No.5-F1-3-failure | / | / |
| No.6 | No.6-F1-1-success | No.6-F2-1-success | No.6-F3-1-success |
|  |  |  | No.6-F3-2-success |
|  |  | No.6-F2-2-success | No.6-F3-1-success |
|  |  |  | No.6-F3-2-failure |
|  | No.6-F1-2-failure | / | / |
|  | No.6-F1-3-failure | / | / |
| No.7 | No.7-F1-1-success | No.7-F2-1-success | No.7-F3-1-success |
|  |  | No.7-F2-2-failure | / |
|  | No.7-F1-2-failure | / | / |
|  | No.7-F1-3-failure | / | / |
| No.8 | No.8-F1-1-success | No.8-F2-1-success | No.8-F3-1-success |
|  |  |  | No.8-F3-2-success |
|  |  | No.8-F2-2-success | No.8-F3-1-success |
|  | No.8-F1-2-success | No.8-F2-1-failure | / |
|  |  | No.8-F2-2-failure | / |
|  | No.8-F1-3-failure | / | / |
|  | No.8-F1-4-failure | / | / |
| No.9 | No.9-F1-1-success | No.9-F2-1-success | No.9-F3-1-success |
|  |  |  | No.9-F3-2-success |
|  | No.9-F1-2-failure | / | / |
|  | No.9-F1-3-failure | / | / |
|  | No.9-F1-4-failure | / | / |
| No.10 | No.10-F1-1-success | No.10-F2-1-success | No.10-F3-1-success |
|  |  |  | No.10-F3-2-success |
|  |  | No.10-F2-2-success | No.10-F3-1-success |
|  |  |  | No.10-F3-2-failure |
|  | No.10-F1-2-success | No.10-F2-1-failure | / |
|  | No.10-F1-3-failure | / | / |
|  | No.10-F1-4-failure | / | / |
|  | No.10-F1-5-failure | / | / |
| No.11 | No.11-F1-1-success | No.11-F2-1-success | No.11-F3-1-success |
|  |  | No.11-F2-2-success | No.11-F3-1-success |
|  | No.11-F1-2-success | No.11-F2-1-success | No.11-F3-1-success |
|  |  |  | No.11-F3-2-failure |
|  |  | No.11-F2-2-failure | / |
|  | No.11-F1-3-success | No.11-F2-1-success | No.11-F3-1-success |
|  | No.11-F1-4-failure | / | / |
|  | No.11-F1-5-failure | / | / |
| No.12 | No.12-F1-1-success | No.12-F2-1-success | No.12-F3-1-success |
|  |  |  | No.12-F3-2-success |
|  |  |  | No.12-F3-3-failure |
|  | No.12-F1-2-failure | / | / |
|  | No.12-F1-3-failure | / | / |
|  | No.12-F1-4-failure | / | / |
| No.13 | No.13-F1-1-success | No.13-F2-1-success | No.13-F3-1-success |
|  |  |  | No.13-F3-2-failure |
|  |  | No.13-F2-2-failure | / |
|  | No.13-F1-2-failure | / | / |
|  | No.13-F1-3-failure | / | / |
|  | No.13-F1-4-failure | / | / |
|  | No.13-F1-5-failure | / | / |
| No.14 | No.14-F1-1-success | No.14-F2-1-success | No.14-F3-1-success |
|  |  |  | No.14-F3-2-success |
|  |  | No.14-F2-2-success | No.14-F3-1-success |
|  | No.14-F1-2-failure | / | / |
|  | No.14-F1-3-failure | / | / |
|  | No.14-F1-4-failure | / | / |
| No.15 | No.15-F1-1-success | No.15-F2-1-success | No.15-F3-1-success |
|  |  |  | No.15-F3-2-success |
|  |  | No.15-F2-2-success | No.15-F3-1-success |
|  | No.15-F1-2-failure | / | / |
|  | No.15-F1-3-failure | / | / |
|  | No.15-F1-4-failure | / | / |
| No.16 | No.16-F1-1-success | No.16-F2-1-success | No.16-F3-1-success |
|  |  |  | No.16-F3-2-success |
|  |  | No.16-F2-2-success | No.16-F3-1-success |
|  |  | No.16-F2-3-failure | / |
|  | No.16-F1-2-failure | / | / |
|  | No.16-F1-3-failure | / | / |
|  | No.16-F1-4-failure | / | / |
| No.17 | No.17-F1-1-success | No.17-F2-1-success | No.17-F3-1-success |
|  |  | No.17-F2-2-failure | / |
|  | No.17-F1-2-failure | / | / |
|  | No.17-F1-3-failure | / | / |
|  | No.17-F1-4-failure | / | / |
| No.18 | No.18-F1-1-success | No.18-F2-1-success | No.18-F3-1-success |
|  |  |  | No.18-F3-2-success |
|  |  | No.18-F2-2-failure | / |
|  | No.18-F1-2-failure | / | / |
|  | No.18-F1-3-failure | / | / |
|  | No.18-F1-4-failure | / | / |
|  | No.18-F1-5-failure | / | / |
| No.19^#^ | No.19-F1-1-success | No.19-F2-1-success^#^ | No.19-F3-1-success^#^ |
|  |  |  | No.19-F3-2-success |
|  |  | No.19-F2-2-success | No.19-F3-1-success |
|  |  | No.19-F2-3-failure | / |
|  | No.19-F1-2-failure | / | / |
|  | No.19-F1-3-failure | / | / |
|  | No.19-F1-4-failure | / | / |
| No.20 | No.20-F1-1-success | No.20-F2-1-success | No.20-F3-1-success |
|  |  | No.20-F2-2-failure | / |
|  | No.20-F1-2-failure | / | / |
|  | No.20-F1-3-failure | / | / |
| No.21 | No.21-F1-1-success | No.21-F2-1-success | No.21-F3-1-success |
|  |  | No.21-F2-2-success | No.21-F3-1-success |
|  |  | No.21-F2-3-failure | / |
|  | No.21-F1-2-failure | / | / |
|  | No.21-F1-3-failure | / | / |
|  | No.21-F1-4-failure | / | / |
|  | No.21-F1-5-failure | / | / |
| No.22 | No.22-F1-1-success | No.22-F2-1-success | No.22-F3-1-success |
|  |  |  | No.22-F3-2-success |
|  |  |  | No.22-F3-3-failure |
|  | No.22-F1-2-failure | / | / |
|  | No.22-F1-3-failure | / | / |
|  | No.22-F1-4-failure | / | / |

^*^ The sample of F0, F2- and F3-PDX cervical cancer xenografts were subjected to Whole-Genome Sequencing (WGS).

^#^ The sample of F0, F2- and F3-PDX cervical cancer xenografts were subjected to Whole-Exome Sequencing (WES).
