# Supplementary material for: Establishment and genetically characterization of patient-derived xenograft models of cervical cancer
Source: BMC Med Genomics. 2022 Sep 8;15:191. doi: 10.1186/s12920-022-01342-5 (PMC9461207; doi:10.1186/s12920-022-01342-5)
Supplement: Supplementary file 2 — Additional file 2. Table S2. The somatic mutation information of the top ten mutated genes. [file 12920_2022_1342_MOESM2_ESM.docx]

Supplementary table 2: The somatic mutation information of the top ten mutated genes

| Gene_symbol | Gene_id | NCBI_Build | Chromosome | Position | Strand | Variant_Classification | Variant_Type | Sample |
| --- | --- | --- | --- | --- | --- | --- | --- | --- |
| PLCB1 | 23236 | GRCh37 | chr20 | 8713973 | + | Missense_Mutation | SNP | {F0} |
| PLCB1 | 23236 | GRCh37 | chr20 | 8722135 | + | Missense_Mutation | SNP | {F0} |
| PLCB1 | 23236 | GRCh37 | chr20 | 8713973 | + | Missense_Mutation | SNP | {F2} |
| PLCB1 | 23236 | GRCh37 | chr20 | 8722135 | + | Missense_Mutation | SNP | {F2} |
| PLCB1 | 23236 | GRCh37 | chr20 | 8713973 | + | Missense_Mutation | SNP | {F3} |
| PLCB1 | 23236 | GRCh37 | chr20 | 8722135 | + | Missense_Mutation | SNP | {F3} |
| KMT2D | 8085 | GRCh37 | chr12 | 49442444 | + | Nonsense_Mutation | SNP | {F0} |
| KMT2D | 8085 | GRCh37 | chr12 | 49442444 | + | Nonsense_Mutation | SNP | {F2} |
| KMT2D | 8085 | GRCh37 | chr12 | 49442444 | + | Nonsense_Mutation | SNP | {F3} |
| LRP1B | 53353 | GRCh37 | chr2 | 141457930 | + | Missense_Mutation | SNP | {F0} |
| LRP1B | 53353 | GRCh37 | chr2 | 141457930 | + | Missense_Mutation | SNP | {F2} |
| LRP1B | 53353 | GRCh37 | chr2 | 141457930 | + | Missense_Mutation | SNP | {F3} |
| NAV3 | 89795 | GRCh37 | chr12 | 78583906 | + | Frame_Shift_Del | DEL | {F0} |
| NAV3 | 89795 | GRCh37 | chr12 | 78583906 | + | Frame_Shift_Del | DEL | {F2} |
| NAV3 | 89795 | GRCh37 | chr12 | 78583906 | + | Frame_Shift_Del | DEL | {F3} |
| TP53 | 7157 | GRCh37 | chr17 | 7577142 | + | Nonsense_Mutation | SNP | {F0} |
| TP53 | 7157 | GRCh37 | chr17 | 7577142 | + | Nonsense_Mutation | SNP | {F2} |
| TP53 | 7157 | GRCh37 | chr17 | 7577142 | + | Nonsense_Mutation | SNP | {F3} |
| MKI67 | 4288 | GRCh37 | chr10 | 129902330 | + | Missense_Mutation | SNP | {F0} |
| MKI67 | 4288 | GRCh37 | chr10 | 129902330 | + | Missense_Mutation | SNP | {F2} |
| MKI67 | 4288 | GRCh37 | chr10 | 129902330 | + | Missense_Mutation | SNP | {F3} |
| FAT1 | 2195 | GRCh37 | chr4 | 187532921 | + | Missense_Mutation | SNP | {F0} |
| FAT1 | 2195 | GRCh37 | chr4 | 187532921 | + | Missense_Mutation | SNP | {F2} |
| FAT1 | 2195 | GRCh37 | chr4 | 187532921 | + | Missense_Mutation | SNP | {F3} |
| PKHD1L1 | 93035 | GRCh37 | chr8 | 110460581 | + | Missense_Mutation | SNP | {F0} |
| PKHD1L1 | 93035 | GRCh37 | chr8 | 110460581 | + | Missense_Mutation | SNP | {F2} |
| PKHD1L1 | 93035 | GRCh37 | chr8 | 110460581 | + | Missense_Mutation | SNP | {F3} |
| SPAG17 | 200162 | GRCh37 | chr1 | 118634274 | + | Missense_Mutation | SNP | {F0} |
| SPAG17 | 200162 | GRCh37 | chr1 | 118634274 | + | Missense_Mutation | SNP | {F2} |
| SPAG17 | 200162 | GRCh37 | chr1 | 118634274 | + | Missense_Mutation | SNP | {F3} |
| KIAA1109 | 84162 | GRCh37 | chr4 | 123109185 | + | Nonsense_Mutation | SNP | {F0} |
| KIAA1109 | 84162 | GRCh37 | chr4 | 123109185 | + | Nonsense_Mutation | SNP | {F2} |
| KIAA1109 | 84162 | GRCh37 | chr4 | 123109185 | + | Nonsense_Mutation | SNP | {F3} |
